# Supplementary material for: Association between oral microbiome and seven types of cancers in East Asian population: a two-sample Mendelian randomization analysis
Source: Front Mol Biosci. 2023 Nov 21;10:1327893. doi: 10.3389/fmolb.2023.1327893 (PMC10702768; doi:10.3389/fmolb.2023.1327893)
Supplement: Supplementary file 1 [file DataSheet1.ZIP › Supplementary Materials/MR plots for tongue/tongue═╝/Ovarian cancer/pheno.2207_to_ovarian cancer_forest.pdf]

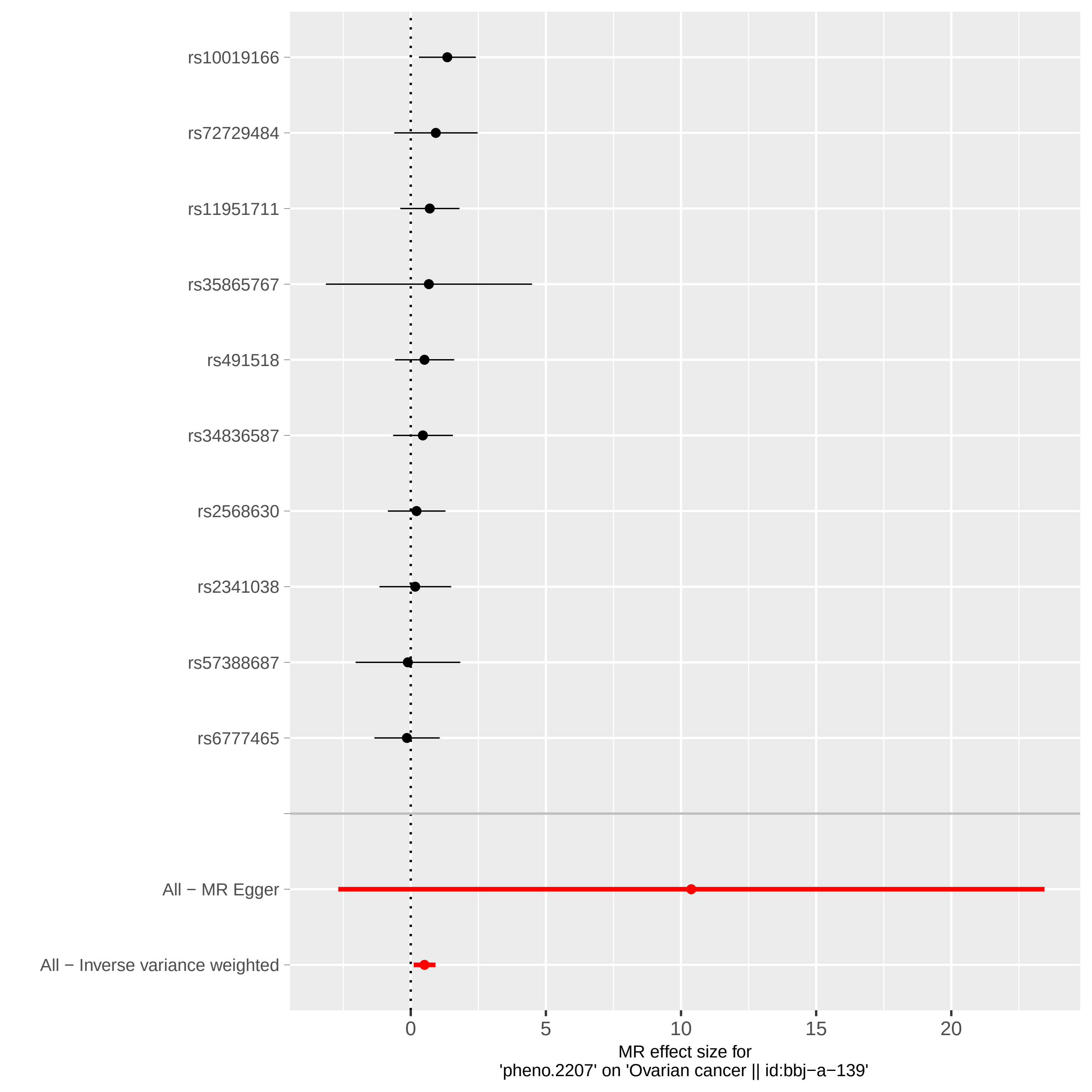
rs10019166
rs72729484
rs11951711
rs35865767
rs491518
rs34836587
rs2568630
rs2341038
rs57388687
rs6777465
All − MR Egger
All − Inverse variance weighted
0
5
10
15
20
MR effect size for
'pheno.2207' on 'Ovarian cancer || id:bbj−a−139'
